# Solving the :: Fusion Nomenclature Challenge for File and Directory Naming

**DOI:** 10.1016/j.jmoldx.2025.08.002

**Published:** 2025-09-01

**Authors:** Tom Bisson, Stefan Kaluziak, Norman Zerbe, Jochen K. Lennerz

**Affiliations:** ∗ Institute of Medical Informatics, Charité – Universitätsmedizin Berlin, Corporate Member of Freie Universität Berlin and Humboldt Universität zu Berlin, Berlin, Germany; † Institute of Pathology, Charité – Universitätsmedizin Berlin, Corporate Member of Freie Universität Berlin and Humboldt Universität zu Berlin, Berlin, Germany; ‡ Department of Pathology, Massachusetts General Hospital, Boston, Massachusetts; § Institute of Pathology, University Hospital RWTH Aachen, Aachen, Germany; ¶ BostonGene, Waltham, Massachusetts

## Abstract

Following recommendations from various consortia and professional societies, the double colon symbol (::) has become an integral part of gene fusion nomenclature (eg, *EML4::ALK*). Although widely adopted, its use presents technical challenges, as many common operating systems restrict the use of colons (:) in file and directory names. Consequently, the double colon (::) is often replaced with an underscore (_) or other allowed characters, introducing ambiguity and inconsistency. The first objective of this work is to raise awareness of this issue. The second objective is to propose a simple technical solution, that is, replacing the command-carrying symbol with a visually similar and functionally distinct ASCII character. The technical solution includes confirmation of functional compatibility and visual compliance with the established fusion nomenclature. The proposal also includes using Unicode characters to replace the slash (/) for alternative variants, the greater than symbol (>) for substitutions, and the asterisk (∗) for nonsense variants, for example. To support consistency, keyboard shortcuts or custom scripts may be used to automate these substitutions. The straightforward solution presented in this paper counterbalances unintended technical consequences and thereby promotes harmonization towards a unified genomic variant nomenclature.

The use of the double colon (::) symbol has been adopted in gene fusion nomenclature to enhance clarity and precision in representing genomic events, as recommended by internationally accepted guidelines.^1^^,^^2^ However, this notation poses practical challenges in file and directory naming because the colon (:) is reserved for special functions in commonly used operating systems, creating a barrier to consistent use and data management. There is currently no solution that preserves the integrity of the nomenclature while ensuring compatibility across all major operating systems.

The double colon (::) symbol has been integrated into gene fusion nomenclature following recommendations by the International System for Human Cytogenomic Nomenclature, Human Genome Variation Society (HGVS), and Variant Interpretation for Cancer Consortium. This notation aligns with cytogenetic conventions and provides immediate recognition of fusion events (eg, *EML4::ALK)*, differentiating these events from other variants. Additionally, this unique notation supports concatenation for complex events (eg, *SYMBOL::SYMBOL/SYMBOL::SYMBOL*) and is instrumental in distinguishing functional fusions from chimeric transcripts. Despite its clarity and widespread acceptance in genomics, the computational adoption of the double colon (::) can present technical obstacles in certain use cases.

This work raises awareness by describing the problem and offers a practical solution to enable compliance with the HGVS Nomenclature across operating systems. By combining engineering and genomic considerations, the work provides an interdisciplinary approach that can overcome the unintended consequences of a well-intended and needed harmonization effort towards a more standardized HGVS nomenclature. Ultimately, this work supports clearer communication, more reliable data exchange, and improved patient care.

## Materials and Methods

### Description of the Problem

**Figure 1:**
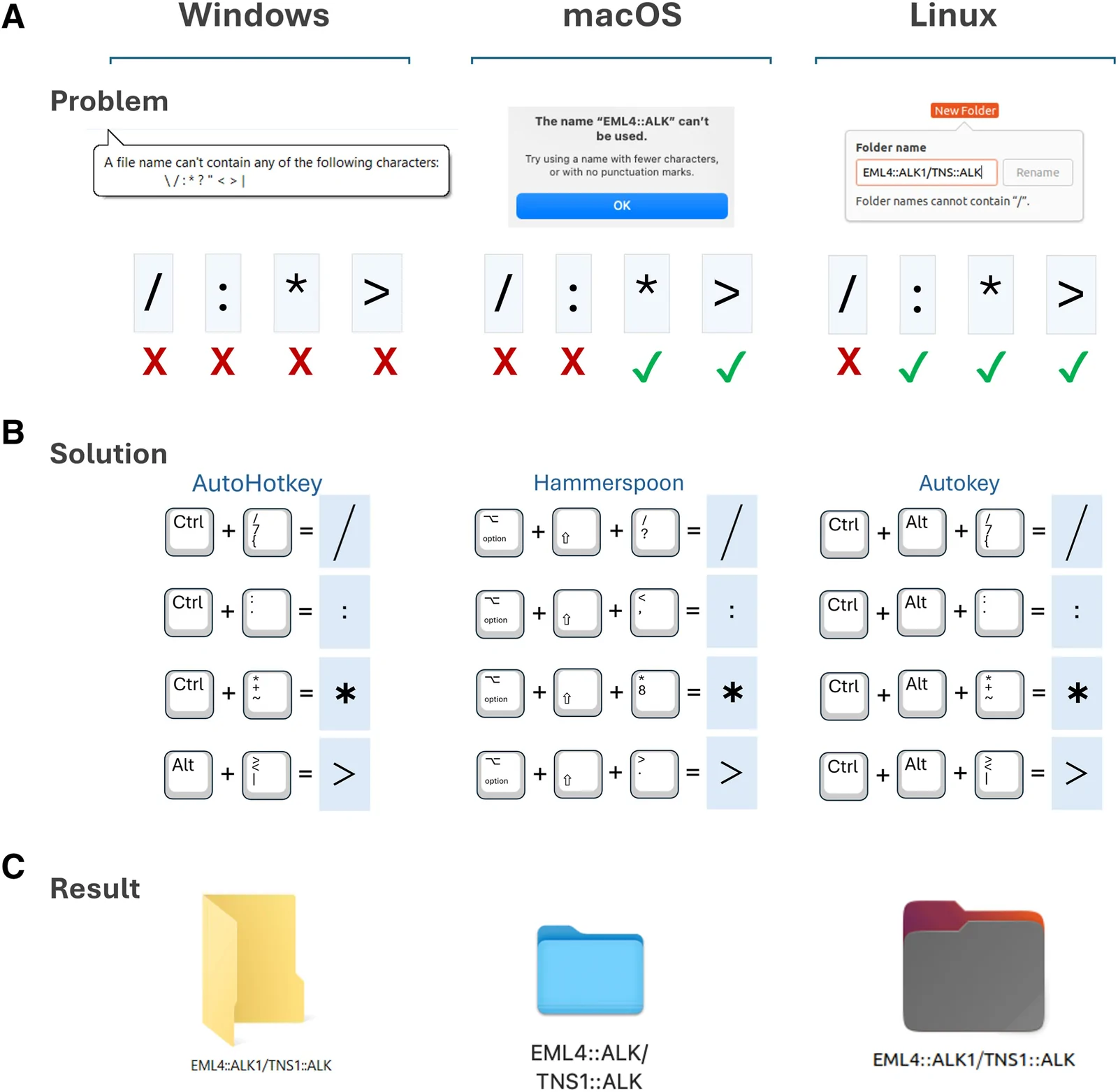
Using double colons (::) in file and directory names according to the HGVS Nomenclature. **A:** Restricted HGVS Nomenclature characters. **Red X symbols** indicate reserved characters; **green checkmarks** indicate characters that are allowed in filenames on the respective operating system**. B:** Solutions to comfortably integrate Unicode replacement characters into custom directory-level workflows. **C:** File and directory names with Unicode replacement characters.

The double colon (::), while clear and intuitive in written scientific contexts, is one of several restricted characters in most operating systems. Figure 1A illustrates the resulting error messages shown when they are used in file or directory names. To overcome this incompatibility between the HGVS Nomenclature and operating systems, a common workaround is to replace these characters (eg, the double-colon with an underscore: *EML4_ALK*), to enable larger analyses across directories or file and folder structures. Simply put, the proposed harmonized nomenclature collides with the inert properties of the operating systems.

### Operating System Constraints

On Windows 10 (Microsoft, Redmont, WA), the colon (:) is reserved for path definitions (eg, C:∖), rendering the double colon (::) incompatible with its file naming conventions because the system interprets the first colon (:) as part of a disk drive name, preceded by a letter and followed by a backslash (∖). Linux (Ubuntu 22.04; Canonical, London, UK) and macOS (Sonoma 14.1.1; Apple, Cupertino, CA) are both compatible with the Portable Operating System Interface, which generally allows the colon (:), but reserves the slash (/) for directory structures. However, on macOS, the colon (:) is still reserved to preserve backwards compatibility with older file systems. While practically possible on Linux, the colon (:) should be used with caution, as it is commonly used as a separator in environment paths, addresses, and protocols, rendering its functional meaning ambiguous (eg, address *https://www.jmdjournal.org*, last accessed May 30, 2025, versus MAC address AC:DE:1F:DF:17:1B). On macOS, it is generally possible to use a backslash (/) as an escape character prior to the reserved characters to allow their use in file and directory names. Yet this still necessitates a distinction between the escaped and reserved characters in automated analyses. Replacing these characters with unreserved alternatives resolves these compatibility issues and ensures precise interpretation while reducing the risk of ambiguity.

### Technical Cross-Platform Solution

The HGVS Nomenclature includes four distinct characters that are reserved in at least one of the three major operating systems and can be replaced with alternative Unicode representations. While typing Unicode characters manually can be tedious, they can be inserted efficiently using hotkey configuration software, as shown in Figure 1. On Windows, AutoHotkey scripts can be used to assign Unicode characters to hotkeys. On macOS, this can be achieved through a tool for automation (Hammerspoon). On Ubuntu, Autokey provides similar functionality by mapping Unicode characters to user-defined key combinations. Cross-platform compatibility was assessed by renaming files and folders and sharing these across platforms. Detailed instructions are provided in a GitHub repository (*https://github.com/Tomatenbiss/HGVS_CharacterKit*, last accessed May 30, 2025).

## Results

A visually similar Unicode character is proposed as a substitute for the double colon (::) to resolve this issue in file and directory names used in genomics workflows. Unicode offers a variety of alternatives with minimal differences in size, shape, and positioning. Selecting replacements characters that are slightly larger and more prominent while maintaining close visual resemblance ensures easy differentiation when placed alongside the original colon (:).

Examination included additional reserved characters defined in the HGVS Nomenclature. Alternative variants in gene fusions are described using a slash (/), substitutions are described using a greater than symbol (>), and nonsense (stop-codon) variants are described using an asterisk (∗). One approach to addressing these issues is to use additional Unicode characters as substitutes for these reserved symbols.

Table 1 presents workable alternatives that resemble the original characters and are confirmed to not be restricted by most common operating systems. Specifically, testing by sharing renamed files and folders confirmed that this article’s approach worked seamlessly on Windows 10, macOS Sonoma, and Ubuntu 22.04 without affecting compatibility or readability.

**Table 1:**
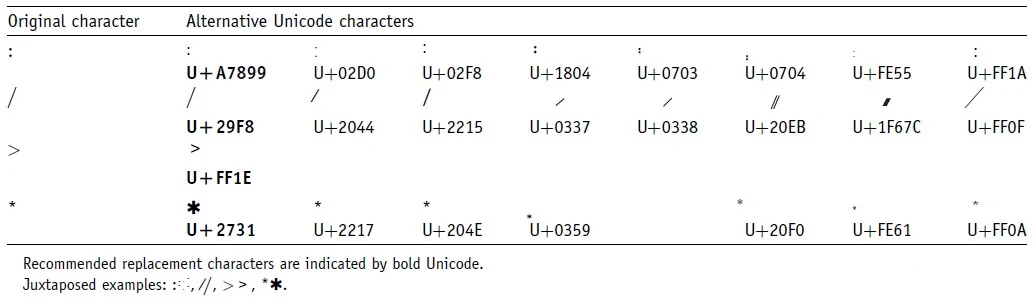
Alternative Unicode Characters for HGVS Notations

As a practical implementation, keyboard shortcuts or custom scripts can automate these substitutions for consistency (Figure 1B). Setup and workflow for these shortcuts and substitutions are described in the GitHub repository accompanying this article (*https://github.com/Tomatenbiss/HGVS_CharacterKit*, last accessed May 30, 2025). The confirmation of successful implementation and cross-platform consistency of these substitutions across three operating systems is provided in Figure 1C.

## Discussion

The presented work addresses an unusual problem and provides a practical solution. Briefly, the proposed HGVS Nomenclature for gene fusions collides with basic functionalities in common operating systems, for example by the double colon (::). A workaround using substitution characters offers a simple, practical solution that enables file and directory names to be aligned with HGVS fusion nomenclature. First, this preserves visual consistency with formal nomenclature, improving traceability and reducing transcription errors. Second, it facilitates direct mapping between files and clinical annotations generated by HGVS-compliant tools. Third, it supports cross-team interoperability in environments where fusion names are frequently referenced across bioinformatic pipelines, reports, and directories. Fourth, the provided solution integrates seamlessly into routine computer use by assigning safe, system-compatible hot keys.

Although Unicode substitutions are not formally recognized by HGVS, they offer a pragmatic compromise between technical constraints and semantic conformity. They resolve a real technical constraint rooted in operating system filename rules, without substantially undermining the visual structure of HGVS notation.

Aside from operating system restrictions, some of these characters can pose security vulnerabilities, depending on how they are handled in computational workflows. For example, characters such as the double colon (::), while syntactically valid in fusion nomenclature, may conflict with scope resolution operators in languages such as C++ and Rust, particularly when fusion names are parsed as strings or passed to external tools. Insufficient input validation may allow arbitrary, potentially harmful code to be executed, sensitive files to be exposed, or data and systems to be compromised. Furthermore, if a script accepts file names from user input and performs operations on them, a filename containing a string with a period slash (./) could allow unauthorized access to parent directories and critical system files: a classic path traversal attack.^3^ In Structured Query Language (SQL), some of these characters, while not inherently dangerous, can increase the impact of injection attacks, which may bypass access controls, retrieve sensitive data such as usernames and passwords, or even delete records.^4^ Although good coding practice can mitigate these risks, developers and scientists without proper programming training may not always be aware of such threats and may involuntarily introduce or exacerbate them.

The proposed solution is simple and practical. Keyboard shortcuts allow users to insert these Unicode characters without memorizing specific codes (eg, Alt + code on Windows;  + code under macOS; or Ctrl + Shift + code under Linux) or relying on copy and paste. Although alternative encodings for visually similar characters are sometimes discouraged due to potential loss of interoperability,^5^ their use in file and directory names is practical and improves usability. Syntactic changes to the official HGVS Nomenclature introduce challenges for interoperability and compatibility but have also been reported as beneficial in other contexts.^6^ As the field is striving towards harmonized genomic variant nomenclature, an official update to the HGVS Nomenclature that incorporates these substitutions would help standardize variant representation across both scientific and computational environments.

## Disclosure Statement

J.K.L. is employed by BostonGene, Waltham, MA.
